# An Attenuated Vaccine Virus of the Neethling Lineage Protects Cattle against the Virulent Recombinant Vaccine-like Isolate of the Lumpy Skin Disease Virus Belonging to the Currently Established Cluster 2.5

**DOI:** 10.3390/vaccines12060598

**Published:** 2024-05-30

**Authors:** Irina Shumilova, Kseniya Shalina, Mohammad Abed Alhussen, Pavel Prutnikov, Alena Krotova, Olga Byadovskaya, Larisa Prokhvatilova, Ilya Chvala, Alexander Sprygin

**Affiliations:** Federal Center for Animal Health, 600901 Vladimir, Russia

**Keywords:** lumpy skin disease, vaccine, Neethling, live-attenuated vaccine, Mongolia/2021, recombinant strain

## Abstract

Lumpy skin disease (LSD) is an emerging transboundary and highly infectious viral disease mainly affecting cattle. The fact that it was initially confined to Africa and then spread beyond its geographical range to other regions, including the Middle East, Turkey, Europe, the Balkans, Russia and Asia, is an indication of the underestimation and neglect of this disease. Vaccination is considered the most effective way to control the spread of LSDV, when combined with other control measures. LSD is now on the rise in Southeast Asia, where the circulating virus belongs to recombinant lineage 2.5. In this study, we evaluated the efficacy of an attenuated LSDV strain belonging to the Neethling cluster 1.1 by challenge with a virulent recombinant vaccine-like LSDV isolate “Mongolia/2021” belonging to cluster 2.5. Some of the vaccinated animals showed an increase in body temperature of 1–1.5 °C above the physiological norm, without clinical signs, local reactions, vaccine-induced viremia or generalization, demonstrating the efficacy and safety of the vaccine strain against a recombinant strain. Furthermore, all the vaccinated animals showed strong immune responses, indicating a high level of immunogenicity. However, the control group challenged with “Mongolia/2021” LSD showed moderate to severe clinical signs seen in an outbreak, with high levels of virus shedding in blood samples and nasal swabs. Overall, the results of the present study demonstrate that the attenuated LSDV Neethling strain vaccine has a promising protective phenotype against the circulating strains, suggesting its potential as an effective tool for the containment and control of LSD in affected countries from Southeast Asia.

## 1. Introduction

The lumpy skin disease virus (LSDV) is recognized as a transboundary and emerging pathogen that inflicts considerable losses on farms and countries where the disease occurs [1]. The etiological agent belongs to the genus *Capripoxvirus* which also includes sheep pox virus and goat pox virus [2]. These viruses share up to 96% nucleotide identity among each other and their names reflect the hosts from which the pathogens were originally recovered [2].

Based on the available genomic data, the LSDV exists as a cluster 1.1 lineage including vaccine strains and virulent field isolates found in South Africa prior to 1990, a cluster 1.2 lineage comprising isolates from the Middle East, Balkans, Greece, Turkey and Russia before 2017 and recombinant vaccine-like (RVL) lineages designated currently as clusters 2.1 through 2.6 [3,4].

LSD is currently distributed in distinct pools in countries of Southeast Asia dominated by a cluster of RVL strains 2.5 [3,5,6] and the Indian subcontinent dominated by KSGP-like strains from cluster 1.2 [3,5,6,7]. Historically, LSD outbreaks aggressively spread into Russia, the Balkans and South Europe in 2015–2016, following the emergence of a natural recombination strain between the vaccine Neethling strain and a vaccine KSGP strain as a consequence of the vaccination campaign with a live attenuated Neethling vaccine outside Russia [3,5,6,8]. The first recombinant LSDV spilled over into Russian cattle in 2017 and from 2018 onwards almost every outbreak of LSD in Russia was due to a unique recombinant virus [6]. In 2019, in China, a novel recombinant vaccine-like strain appeared close to the border with Kazakhstan that had never been reported before and was subsequently designated as cluster 2.5 which entrenched in the region with catastrophic consequences for the economy of Southeast Asian countries [3,9,10].

The only efficient way of fighting LSD is through vaccination which can be implemented with heterologous and homologous vaccines [11]. Although homologous vaccines do induce stronger protection in cattle as compared to heterologous vaccines, homologous vaccines are prone to molecular evolution in susceptible hosts and cause unwanted side effects called “Neethling disease” which are exempt from being reported to the World Organization for Animal Health (WOAH), explaining the reason why vaccine-like strains are not reported [12]. Heterologous vaccines demonstrate greater safety due to the inability to replicate in bovine hosts [13,14]. Regardless of this, a search for efficient vaccine preparations is ongoing with a view to eradicating LSD [14,15].

Recent experimental and field studies on homologous vaccines demonstrated potency and efficacy despite the negative side effects they may exert. However, most studies were focused on the protection from cluster 1.2 strains [14], whereas the distribution of cluster 1.2 strains is limited to the Middle East, Africa and Indian Subcontinent, whereas cluster 2.5 strains of recombinant origin are now circulating in China, Thailand, Vietnam, etc. [6,14]. Unfortunately, the efficacy of the commonly used Neethling vaccines is unknown against novel recombinant lineages as well as the currently dominating lineage from Southeast Asia cluster 2.5; however, there is evidence that recombinant vaccine-like strains exhibit novel properties absent from parental strains of cluster 1.1 and cluster 1.2 such as non-vector borne transmission even in freezing months, overwintering in northern latitudes and more aggressive growth in vitro [16,17].

The objective of this study was the evaluation of an LSDV strain from the Neethling cluster 1.1 to confer protection in cattle against the challenge of a virulent recombinant vaccine-like strain from cluster 2.5 Mongolia/2021 when administered intravenously and subcutaneously.

## 2. Materials and Methods

### 2.1. Virus Strains

During this work, we used two LSD virus strains, namely, one as a vaccine strain and the other (field isolate) as an inoculum: (1) The field strain “Mongolia/2021” originated from an outbreak in Mongolia in 2021 [18]. The virus underwent a few passages (fewer than 5 passages) on goat testicular cells for isolation. (2) A vaccine preparation was produced from a vaccine-like isolate Bashkortostan/2017 that had 100% nucleotide identity to Neethling vaccines from the Neethling cluster 1.1 by Sanger sequencing of the RP030 gene [19]. It was passaged 25 times in embryonated chicken eggs, 10 times on sheep ovine cells and 15 times on goat testis cells to achieve an attenuated phenotype.

A series of 10-fold dilutions were prepared and the virus was titrated using a standard plaque assay in 96-microwell plates. The plates were incubated at 37 °C with 5% CO_2_ for 72 h and monitored for a monolayer cytopathic effect (CPE). The negative control wells had to demonstrate the absence of a CPE, whilst characteristic CPEs in the form of lumps on the cell layer had to be present in the wells for the virus or positive control. The virus titer was calculated according to the Spearman–Karber method and is reported as described [20]. The results are expressed in a logarithm as a 50% tissue culture infective dose (log TCID_50_).

### 2.2. Experimental Design

Experiments were carried out using sixteen Russian Black Pied breed bulls (Bos taurus) aged 12–18 months, weighing between 270 and 300 kg in an insect-proof isolation unit with veterinary care. All animals were inspected twice a day by the veterinary staff. Water and feed were provided ad libitum. Animals were housed in the facility for 2 weeks before the onset of the experiment, in order to adapt to the conditions, whilst blood samples and nasal swabs were collected for PCR and neutralization tests to rule out previous or present *Capripoxvirus* exposure.

The animals were then allocated into two groups, each group having six experimental animals (vaccinated) and two sham-vaccinated as the negative controls (Table 1).

On the first day of the experiment (0 days post inoculation—dpi), all animals except the control (SC-C and IV-C) subcutaneously received 1 mL of 4.1 log TCID_50_/_mL_ of the vaccine as designated. Animals were monitored for 21 days through registration of body temperature and clinical changes, blood samples and nasal swabs were collected every second day for PCR analysis, ELISA and virus isolation on Madin–Darby bovine kidney (MDBK) cells according to the method described by Irons et al. [21].

**Figure 1:**
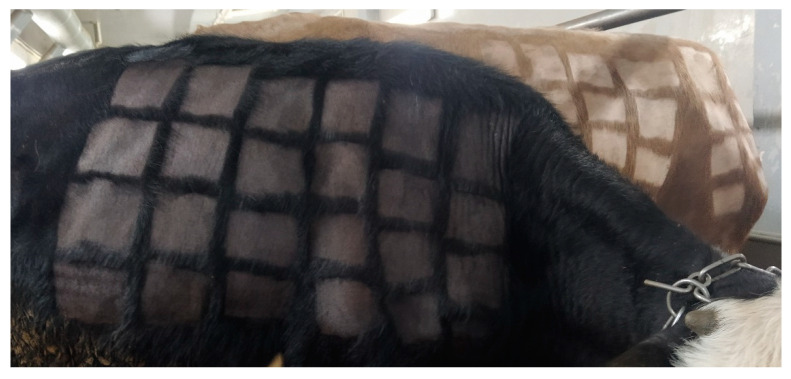
Titration scheme of LSDV “Mongolia/2021” on animals of group 1.

On the 21st dpi all animals of the first group, including the control, received 0.1 mL of 5.5 log TCID_50_/mL of the LSDV field isolate “Mongolia/2021”; the virus was titrated using 10-fold dilutions and injected subcutaneously as shown in Table 2 and Figure 1. Group 2 received 2 mL of 5.5 log TCID_50_/mL of LSDV intravenously.

After the challenge, all animals were observed for a further 28 days until the end of the experiment (until 49 dpi).

The experimental design and the euthanasia protocol were endorsed by the Ethics Committee of the Federal Center for Animal Health, Russia (Permit Number: No. 11/4-29112023) and implemented in compliance with the Directive 2010/63/EU on the protection of animals used for scientific purposes. The euthanasia procedure included captive-bold penetration to stun and render the animals insensible at slaughter, followed by the injection of the muscle relaxant, Adilinum super (Federal Center for Toxicological, Radiation and Biological Safety, Kazan, Russia). The latter is administered at the recommended dose of 5 mg/kg according to the drug use instruction approved by the Russian Federal Service for Veterinary and Phytosanitary Surveillance in 2008. At the recommended dose, the Adilinum mechanism of action results in a rapid and painless death.

### 2.3. Clinical Score

For the whole period of the experiment (49 days), animals were monitored on a daily basis for clinical changes, changes in body temperature and behavior. Body temperatures were measured daily from 0 dpi until 49 dpi (normal range of body temperature prior to experimental infection: 38.5–39.5 °C). For the examination of clinical reactions from 0 dpi to 49 dpi, we used the modified clinical score system of Carn and Kitching [22] as recommended by Wolff et al. [23] (Supplementary Table S1). At the end of the experiment, the respective cattle were euthanized due to ethical reasons. Samples were taken every second day, and Ethylenediaminetetraacetic acid (EDTA) blood and nasal swabs were taken to test for viral presence in blood and virus shedding, respectively. Serum samples were used for the serological evaluation.

### 2.4. PCR Testing

Samples were collected aseptically using sterile polyester swabs to collect nasal secretions from each animal. One swab was used per nostril. Nasal swabs were inserted approximately 15 cm into each nostril and gently rubbed into the nasal mucosal surface; both swabs were collected in a tube with 2 mL of cold phosphate-buffered saline (PBS), then shaken in the PBS, and the fluid was squeezed out of the swab by pressing the swab against the tube wall, and the tubes were placed on ice for transport and further use. A 200 μL aliquot was used for total nucleic acid extraction using the QIAamp DNA Mini Kit (Qiagen, Hilden, Germany) following the manufacturer’s instructions. Sample extracts were analyzed for the presence of LSDV DNA using real-time PCR (qPCR) based on ORF044 as previously described [24].

The fluorogenic probe was labeled at the 5′ end with the FAM reporter dye and with BHQ as a quencher at the 3′ end. Selected primers (df4ln: CAAAAACAATCG-TAACTAATCCA and zdr4ln: TGGAGTTTTTATGTCATCGTC) and probes (zdpro4ln1:Fam-TCGTCGTCGTTTAAAACTGA-BHQ1) were synthesized by Syntol (Moscow, Russia) [24]. PCR was performed using a Rotor-Gene Q (Qiagen, Germany) instrument and the following thermal-cycling profile: 95 °C for 10 min, followed by 45 cycles at 95 °C for 15 s and 60 °C for 60 s. The final reaction volume was 25 μL containing 10 pmol of each primer, as well as 5 pmol of the probe, 5 μL of 25 mM MgCl_2_, 5 μL 5 × PCR Buffer (Promega, Madison, WI, USA), 1 μL of 10 pmol dNTPs (Invitrogen, Carlsbad, CA, USA) and deionized water to make up the final volume. Samples were tested and results were interpreted according to the protocol, as previously described [24].

### 2.5. ELISA Testing

An ELISA-test system “ID Screen^®^ Capripox Double Antigen” Multi-species ID.VET” (ID. Vet, Grabels, France) was used to detect the specific antibodies of *Capripoxvirus* in the serum or plasma of susceptible animals according to the manufacturer’s instructions. Interpretation of the results was conducted as aggregate to positive control ratio S/P percentage (S/P %) using the following formula:

$$S/P\;\%=\frac{\mathrm{sample}\;\mathrm{OD}-\mathrm{negative}\;\mathrm{control}\;\mathrm{OD}}{\mathrm{positive}\;\mathrm{control}\;\mathrm{OD}-\mathrm{negative}\;\mathrm{control}\;\mathrm{OD}}\times 100$$

OD: optical density

The samples with Sp < 30% are considered negative, while samples with SP ≥ 30% are considered positive.

### 2.6. Statistical Analysis

Data were analyzed and graphs were generated using Microsoft Excel 2019. The difference in qPCR Ct values between positive samples was tested using the non-parametric Mann–Whitney U test. A *p* value < 0.05 was considered statistically significant. Serological results were represented as mean ± SE.

## 3. Results

**Figure 2:**
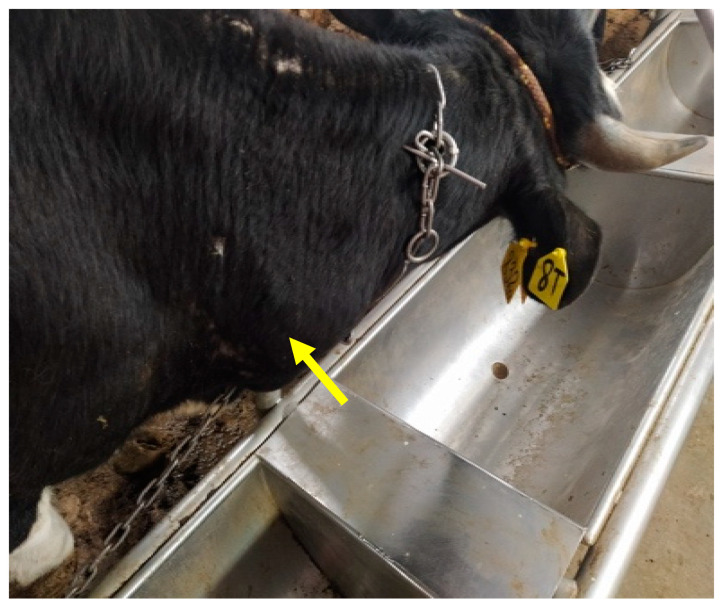
Swelling at the point of vaccination in animal 8-SC.

On the first 21 dpi of the experiment, a slight increase in the body temperature of two vaccinated animals (No. 8-SC and 8-IV) was recorded with an average of 1–1.5 degrees above the physiological norm. Swelling was observed at the site of vaccine administration in the same two animals (Figure 2). The physiological parameters of the remaining animals (vaccinated and control) in both groups were normal.

The qPCR results showed that the genome of the LSDV vaccine was detected in the blood and swabs taken from experimental animals on different days, but by the end of the vaccination period the genome was no longer detected (Table 3).

As presented in Table 3, three animals from the first group (No. 2-SC, 5-SC and 8-SC) had the virus genome detected in their blood: once in the blood taken from No. 2-SC (dpi 10), twice in the blood taken from No. 5-SC (dpi 10–12) and in the blood taken from animal No. 8-SC for three consecutive times between dpi 8 and 12. This animal had an elevated body temperature above normal in the same period, while for animals from group 2, it was detected in No. 3-IV and 8-IV only for one day (12 dpi).

Since the challenge was performed on the 21st dpi using LSDV “Mongolia/2021” and as mentioned in Section 2.2, animals were not monitored for the 6 days, then on the 28th dpi samples were taken and clinical changes were recorded. Also, titration in group 1 was assessed as presented in Table 4.

**Figure 3:**
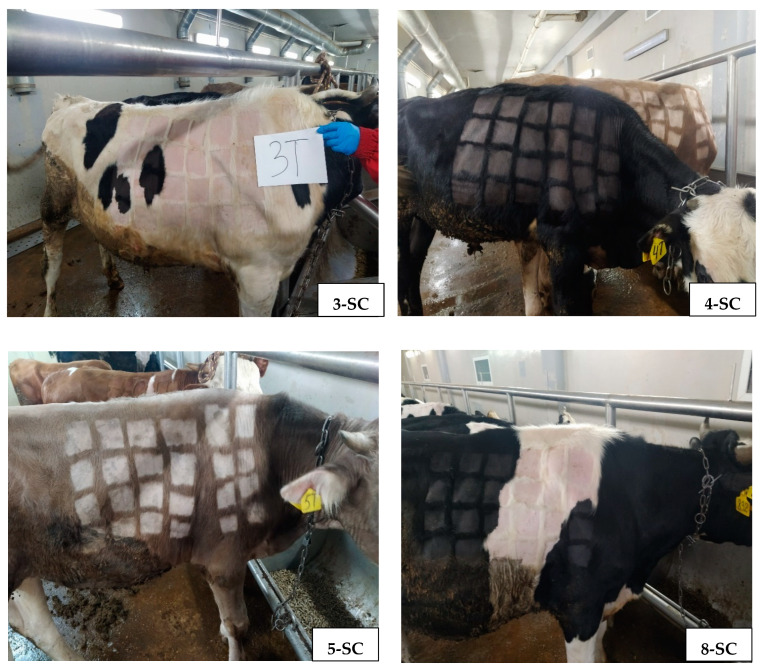
Titration of LSDV subcutaneously in vaccinated animals of group 1, without nodules appearing in shaved areas, where the virus was inoculated.

**Figure 4:**
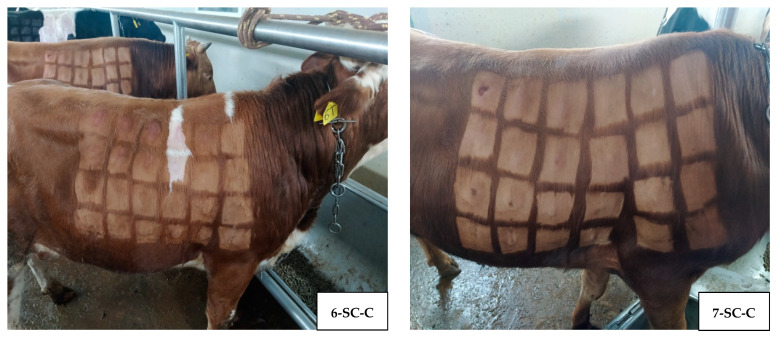
Titration of LSDV subcutaneously in control animals of group 1, with nodules appearing in shaved areas, where the virus was inoculated.

For all experimental animals of group 1 except for the control (No. 6-SC-C and 7-SC-C), no clinical changes were detected on the skin at the place of injection of the LSDV. The results were positive (nodules were detected) when examining control animals, using different titers of the virus (Figure 3 and Figure 4).

**Figure 5:**
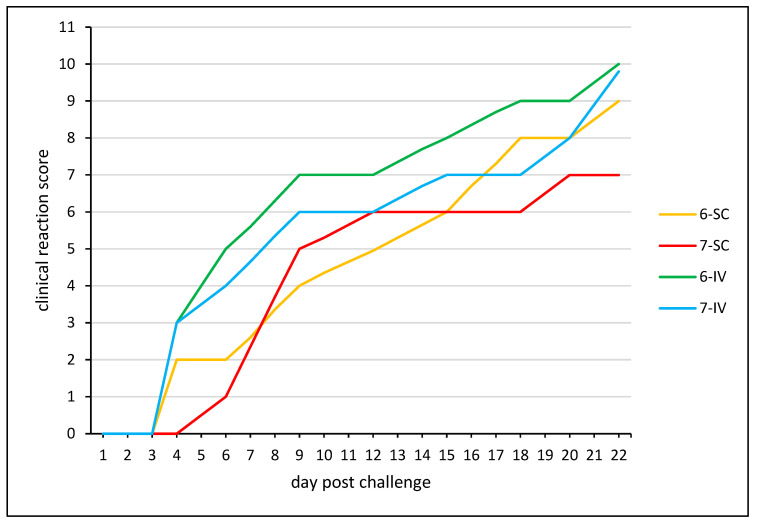
Clinical reaction score of control animals inoculated with infectious LSDV “Mongolia/2021”. Clinical reaction score (CRS) was measured daily from 28 dpi until end of experiment 49 dpi for control groups. CRS = 0 means no clinical signs, CRS 1–5 displays mild clinical course, and CRS 6–10 shows severe clinical reaction (Supplementary Table S1).

No clinical signs were detected in other experimental animals of both groups 1 and 2 (vaccinated), and the qPCR results were also negative until the end of the experiment (49 dpi). On the contrary, control animals, from the 28th dpi, developed a severe clinical course typical for an LSDV infection after inoculation of a virulent challenge virus strain (Figure 5), and the genome of LSDV was detected in the blood and swabs were taken from these animals; viral shedding can also be observed (Table 5).

The following clinical signs were observed in control animals after infection: enlargement of regional lymph nodes on the right side of the body, fever, swelling of the scrotum, erosions on the scrotum and the inguinal region, and multiple nodular skin lesions (3–5 cm) localized mainly on the neck, scapula, flanks, and inguinal region.

According to the results of the observations, the control animals showed a pronounced skin reaction to the virus injection with subsequent development of a generalized form of the disease from days 7–9 (Figure 5).

As presented in Table 5, DNA of the LSDV was detected in all control animals that received the “Mongolia/2021” inoculum (SC-C and IV-C), with significantly higher viremia (*p* < 0.05) in the control animals of the second group (IV-C). The viremia progressed until the end of the experiment.

All control animals showed virus shedding until 49 dpi, which was confirmed by the positive results of their nasal swab samples, with significantly (*p* < 0.05) higher results in the animals of the second control group (IV-C).

**Figure 6:**
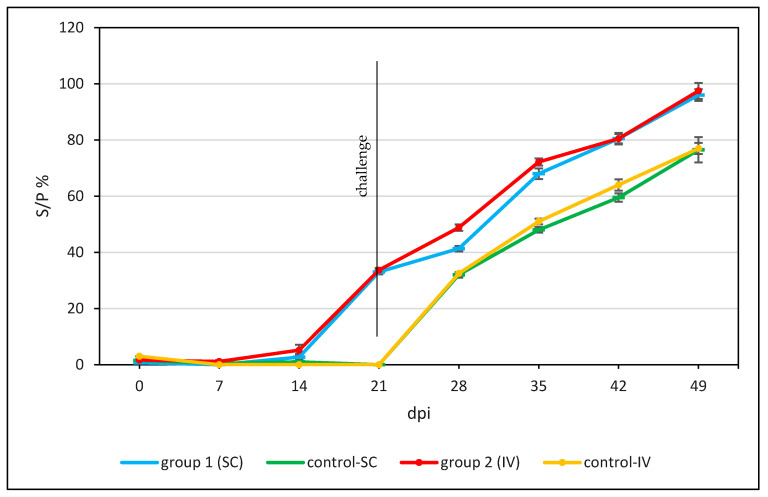
A serological examination of the sera of the experimental and control groups taken during the study using ELISA. The samples were defined as positive at an S/P% ratio ≥ 30. Mean ± SE.

The results of antibody response in all experimental animals are presented in Figure 6.

Our research results show that by using the ELISA, the first positive results in the serological analyses were observed already at 21 dpi (on the day of challenge infection with LSDV “Mongolia/2021”) in all vaccinated animals (Figure 6), with S/P ratios between 31 and 36%, whereas the ELISA was negative in all animals of the control group at 21 dpi, but the 6-SC-C animal of control group 1 remained negative, but a rising antibody level below the cut-off S/P % was detected at 14 dpi (Figure 6).

In the challenge control group (SC, IV), the first serological response was observed at 28 dpi (7 days after inoculation). After the challenge infection, the S/P% ratio increased over time in almost all animals and continued to increase after inoculation, reaching a peak at the 49th dpi. The level of the antibody response was stronger and more rapid in both vaccinated groups than in the controls, regardless of the method of inoculation. These results indicate that vaccinated animals developed a rapid and robust immune response compared to unvaccinated control animals.

**Figure 7:**
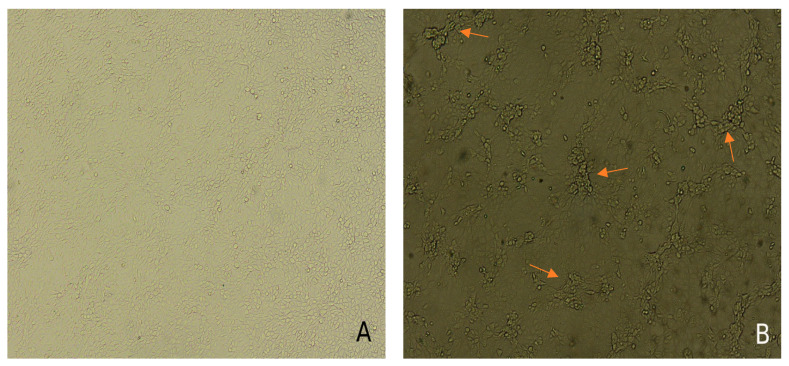
Isolation of LSDV on Madin–Darby bovine kidney (MDBK) cells. (**A**) Normal non-inoculated MDBK cells (negative control). (**B**) Cytopathic effect of LSDV on MDBK cells 4 days post-inoculation with LSDV “Mongolia/2021” obtained from kidney cells of 7-IV-C animal (10× magnification). The arrows indicate the aggregation, cell rounding and degeneration.

The LSDV pathogen was efficiently isolated and adapted on (MDBK) cells from nasal swabs and other pathological materials obtained from the control animals of both groups (SC-C and IV-C) inoculated with LSDV “Mongolia/2021”, which showed aggregation, cell rounding and degeneration, whereas no viable LSDV pathogen was isolated from the other experimental animals (vaccinated), during the 49 days of the experiment (Figure 7).

## 4. Discussion

Lumpy skin disease (LSD) is no longer restricted to the African continent, where it is considered an endemic disease but has spread to other regions beyond its geographical range, including the Middle East, Turkey, Europe, the Balkans, Russia and Asia [6,19,25,26]. It has been listed by WOAH as one of the most reportable transboundary viral animal diseases causing significant economic losses [27,28].

The vaccination of all susceptible cattle, in combination with other disease control interventions, namely stamping out, movement restrictions of animals and vector control, is considered the most effective way to control the spread of LSDV [26,29,30,31]. Several different live-attenuated vaccines have been developed and used to protect cattle against LSDV, including the homologous LSDV Neethling strain [32] and the KSGP O-240 strain [15,33,34], or the heterologous sheep pox RM65 strain [35], the Romanian sheep pox virus strain [36], NISKHI strain [37,38], or others. The use of the live-attenuated Neethling LSDV strain vaccine has been shown to provide protection against the disease during the most recent outbreak of LSD in the Balkan countries [15,31,39].

In this study, we for the first time successfully evaluated the efficacy of the LSDV attenuated strain of the Neethling lineage against a virulent recombinant vaccine-like strain “Mongolia/2021”. Generally, very clear differences in the pathogenesis of the LSDV Neethling vaccine strain and the LSDV “Mongolia/2021” strain were observed in our study. The vaccine strain was well tolerated by the animals without any clinical changes or any other adverse effects on the general health of the animals. Furthermore, some of the vaccinated animals had low viral copy numbers in their blood on days 8–12 dpi, while virus isolation was not detected in the blood of other vaccinated animals (Table 3). These results are consistent with those reported in previous studies [33,38,39,40,41,42].

Our results showed that there was an increase in the temperature of two animals after vaccination on the 5th and 6th dpi, which is usually reported after vaccination of animals with the Neethling strain [33,38,39,40,41,42]; moreover, this increase in temperature after vaccination could also be a result of stress factors, since no specific pattern of fever was observed. However, fever for several days is commonly observed in some animals vaccinated with other LSDV or viral vaccines [30]. Hamdi et al. observed a moderate temperature rise (39.5–39.6 °C) and local reactions a few days after vaccination with an inactivated prototype vaccine, which persisted for several days [40]. Our results are consistent with previously published research describing elevated body temperatures [35,41,42] and local reactions after vaccination [42,43,44,45], but no generalized clinical signs or mortalities [41]. Some Neethling vaccine strains do cause the formation of skin lesions [41,46].

The challenge with the recombinant LSDV “Mongolia/2021” strain resulted in an elevated body temperature in all control animals starting about 4–6 days after inoculation and persisting until the end of the experiment, which is similar to observations after inoculation with a virulent South African field strain [47] and after inoculation with the LSDV-Macedonia 2016 strain [48], whereas no clinical signs were detected in vaccinated animals of both groups and qPCR results were also negative until the end of the experiment. In addition, control animals developed generalized LSDV, and large skin nodules (3–5 cm) were similar to those reported by WOAH (founded as OIE) (2016) [49].

The LSDV genome loads were detected starting from day 7–10 post-inoculation with “Mongolia/2021” in all control animals during the study in blood samples and nasal swabs in these animals; these results are similar to those previously described by [50]. Furthermore, in previous studies, shedding of the LSDV strain Udmurtiya/2019 continued for more than one month in blood samples and nasal swabs, while the strain Saratov/2017 was detected in blood for the same period, but lasted for 27 days in nasal swabs [17]. Our results show a correlation between the viral DNA detection in swab samples, representing virus shedding, and the degree of severity of clinical symptoms, which is consistent with a study by [48]. On the contrary, in a study in 2005, it was shown that there was no correlation between the degree of severity of clinical signs and the duration of viremia after inoculation with a virulent South African field strain of the LSDV [47]. However, the LSDV viral DNA was not detected in the samples from vaccinated animals. Interestingly, this finding contrasts with the results of [45], who successfully re-isolated the LSDV from skin nodules after prophylactic vaccination with Lumpyvax/SIS-Type.

In addition, we have found that the use of nasal swabs provides better sensitivity than the use of blood with EDTA and they are also easy to handle (Table 5). Thus, nasal swab collection appears to be the preferred non-invasive sampling method for use during natural outbreaks; our results are consistent with those reported by Möller et al., who found lower viral loads in EDTA blood, serum and oral swabs compared to nasal swabs [48].

Although seroconversion occurs in most vaccinated animals against *Capripoxvirus* infections, some vaccinated animals may be completely protected without an increase in blood antibody levels [33,51,52], which may be relatively explained by the nature of some cattle breeds or the type of vaccine used [53,54]. Therefore, to evaluate the protection provided by the vaccine, it is essential to conduct challenge studies with a highly virulent field strain [26], and a seronegative LSDV in young dairy cattle [38]. Our results indicated that there was no seropositivity in immunized animals, but after inoculation with LSDV “Mongolia/2021”, antibodies were detected by ELISA from 21 dpi, which is in agreement with a previous study [48]. Furthermore, an increase in antibody levels was observed from the day of infection until 49 dpi, which is analogous to the results of [48,55]. The WOAH Terrestrial Guidelines indicate that antibodies against *Capripoxvirus* can be detected from day 2 after the onset of clinical signs, but a significant increase in antibody titers usually occurs between 21 and 42 days [49]. The level of the antibody response was stronger and more rapid in both vaccinated groups than in the control animals, regardless of the method of inoculation. This requires the development of a test system to differentiate between antibodies produced as a result of infection or vaccination, which is not possible given the existing repertoire of available vaccines [51]. In addition, isolation of LSDV “Mongolia/2021”, obtained from the control animals, on Madin–Darby bovine kidney (MDBK) cells showed a specific cytopathic effect manifested by aggregation, rounding and degeneration of cells, and clustering after the third day post-inoculation. Massive detachments of cells were observed on the 4th day. These results are consistent with those of [56,57].

Therefore, our results are consistent with those of Hamdi et al. who achieved complete clinical protection after vaccination with an inactivated attenuated LSDV Neethling strain [40]. In contrast to another study, animals immunized with an inactivated LSDV were only partially protected against challenge infection [55].

Given the critical function of T cells in providing immunity against poxvirus infections [58], this aspect needs further investigation when vaccine strain evaluations are concerned. This could be accomplished via blood transcriptomics: RNA sequencing offers insights into a cattle’s immunity at the level of immune cells and gene expression [59]. In our study, we did not evaluate the T-cell immune response, which could be a limitation of the work; however, future studies are warranted to shed light on the immune trajectories following infection or vaccination.

In any event, a vaccine to be used in a given territory must be decided based on the pros and cons of any type of vaccine considering all possible consequences.

## 5. Conclusions

In conclusion, our results indicate that the attenuated LSDV vaccine strain of the Neethling cluster 1.1 can protect cattle against challenges with the virulent LSDV field isolate “Mongolia/2021”. Complete clinical protection was observed in all vaccinated animals after challenge infection, without vaccine-related viremia and side effects, while control animals succumbed to infection. In addition, our data indicate that this LSDV strain was able to demonstrate robust immune responses in the vaccinated cattle against a virulent recombinant LSDV isolate.

## Figures and Tables

**Table 1:** Schematic distribution of experimental animals.

Group 1								Group 2							
1-SC	2-SC	3-SC	4-SC	5-SC	6-SC-C	7-SC-C	8-SC	1-IV	2-IV	3-IV	4-IV	5-IV	6-IV-C	7-IV-C	8-IV

SC—subcutaneous injection; SC-C—control animals in the SC group; IV—intravenous injection; IV-C—control animals in the IV group.

**Table 2:** Titration scheme of LSDV “Mongolia/2021” used for challenge on animals.

5.5 lg TCID_50_/mL	4.5 lg TCID_50_/mL	3.5 lg TCID_50_/mL	2.5 lg TCID_50_/mL	1.5 lg TCID_50_/mL	0.5 lg TCID_50_/mL
5.5 lg TCID_50_/mL	4.5 lg TCID_50_/mL	3.5 lg TCID_50_/mL	2.5 lg TCID_50_/mL	1.5 lg TCID_50_/mL	0.5 lg TCID_50_/mL
5.5 lg TCID_50_/mL	4.5 lg TCID_50_/mL	3.5 lg TCID_50_/mL	2.5 lg TCID_50_/mL	1.5 lg TCID_50_/mL	0.5 lg TCID_50_/mL
5.5 lg TCID_50_/mL	4.5 lg TCID_50_/mL	3.5 lg TCID_50_/mL	2.5 lg TCID_50_/mL	1.5 lg TCID_50_/mL	0.5 lg TCID_50_/mL

**Table 3:** qPCR results detecting the LSDV genome in the blood and nasal swabs of animals on the first 21 days of the experiment (Ct values).

dpi	1-SC	2-SC	3-SC	4-SC	5-SC	6-SC-C	7-SC-C	8-SC	1-IV	2-IV	3-IV	4-IV	5-IV	6-IV-C	7-IV-C	8-IV
1	N/N *	N/N	N/N	N/N	N/N	N/N	N/N	N/N	N/N	N/N	N/N	N/N	N/N	N/N	N/N	N/N
3	N/N	N/N	N/N	N/N	N/N	N/N	N/N	N/N	N/N	N/N	N/N	N/N	N/N	N/N	N/N	N/N
5	N/N	N/N	N/N	N/N	N/N	N/N	N/N	N/N	N/N	N/N	N/N	N/N	N/N	N/N	N/N	N/N
7	N/N	N/N	N/N	N/N	N/N	N/N	N/N	N/N	N/N	N/N	N/N	N/N	N/N	N/N	N/N	N/N
8	N/N	N/N	N/N	N/N	N/N	N/N	N/N	39.1/N	N/N	N/N	N/N	N/N	N/N	N/N	N/N	N/N
10	N/N	36.7/N	N/N	N/N	35.1/N	N/N	N/N	38.7/N	N/N	N/N	N/N	N/N	N/N	N/N	N/N	N/N
12	N/N	N/N	N/N	N/N	33.8/N	N/N	N/N	37.1/N	N/N	N/N	36.7/N	N/N	N/N	N/N	N/N	35.0/N
14	N/N	N/N	N/N	N/N	N/N	N/N	N/N	N/N	N/N	N/N	N/N	N/N	N/N	N/N	N/N	N/N
16	N/N	N/N	N/N	N/N	N/N	N/N	N/N	N/N	N/N	N/N	N/N	N/N	N/N	N/N	N/N	N/N
18	N/N	N/N	N/N	N/N	N/N	N/N	N/N	N/N	N/N	N/N	N/N	N/N	N/N	N/N	N/N	N/N
20	N/N	N/N	N/N	N/N	N/N	N/N	N/N	N/N	N/N	N/N	N/N	N/N	N/N	N/N	N/N	N/N
21	N/N	N/N	N/N	N/N	N/N	N/N	N/N	N/N	N/N	N/N	N/N	N/N	N/N	N/N	N/N	N/N

N—negative qPCR result (genome not detected); *—results of nasal swabs; Ct < 38 positive; Ct = 38–39–99 doubtful.

**Table 4:** Titration results of LSDV “Mongolia/2021” on control animals of group 1 in accordance with Table 2.

7-SC-C						6-SC-C					
5.5 lg	4.5 lg	3.5 lg	2.5 lg	1.5 lg	0.5 lg	5.5 lg	4.5 lg	3.5 lg	2.5 lg	1.5 lg	0.5 lg
+	+	+	+	−	−	+	+	+	+	+	+
+	+	+	+	+	−	+	+	+	+	+	+/−
+	+	+	+	+	−	+	+	+	+	+	−
+	+	+	+	−	−	+	+	+	+	+	+

“+”—skin reaction at the site of virus inoculation; “−”—no skin reaction; “+/−”—skin reaction is not clearly expressed.

**Table 5:** The qPCR results detecting the LSDV genome in the blood and nasal swabs of animals from the 28th dpi until the end of the experiment (Ct values).

dpi	1-SC	2-SC	3-SC	4-SC	5-SC	6-SC-C	7-SC-C	8-SC	1-IV	2-IV	3-IV	4-IV	5-IV	6-IV-C	7-IV-C	8-IV
28	N/N	N/N	N/N	N/N	N/N	34.7/N	N/N	N/N	N/N	N/N	N/N	N/N	N/N	N/29.6	34/33.8	N/N
30	N/N	N/N	N/N	N/N	N/N	N/28.3 *	N/30.9	N/N	N/N	N/N	N/N	N/N	N/N	34.4/26.6	32.3/28.9	N/N
32	N/N	N/N	N/N	N/N	N/N	33.4/24.9	32.7/29.9	N/N	N/N	N/N	N/N	N/N	N/N	30.8/22.2	26.8/26.1	N/N
34	N/N	N/N	N/N	N/N	N/N	33.5/28.7	29.6/27.2	N/N	N/N	N/N	N/N	N/N	N/N	27.1/21.5	27.5/29.9	N/N
36	N/N	N/N	N/N	N/N	N/N	29.8/25.4	24.9/30.4	N/N	N/N	N/N	N/N	N/N	N/N	27.7/29.9	24. 9/27.7	N/N
38	N/N	N/N	N/N	N/N	N/N	28.4/22.2	25.7/25.7	N/N	N/N	N/N	N/N	N/N	N/N	23.4/19	25.3/18.5	N/N
40	N/N	N/N	N/N	N/N	N/N	25.1/21.3	24.1/24.3	N/N	N/N	N/N	N/N	N/N	N/N	24.4/25.7	21.7/21.7	N/N
42	N/N	N/N	N/N	N/N	N/N	24.7/14.2	23.7/19	N/N	N/N	N/N	N/N	N/N	N/N	23.1/20.1	16.5/24.2	N/N
44	N/N	N/N	N/N	N/N	N/N	20.2/18	24.9/21	N/N	N/N	N/N	N/N	N/N	N/N	20.6/22.3	20.5/22.2	N/N
46	N/N	N/N	N/N	N/N	N/N	23.6/15.3	20.1/19.6	N/N	N/N	N/N	N/N	N/N	N/N	20/17.2	22.3/16.5	N/N
49	N/N	N/N	N/N	N/N	N/N	22.7/14.5	25.2/27.4	N/N	N/N	N/N	N/N	N/N	N/N	19.8/25.5	17.2/14.3	N/N

N—negative qPCR result (genome not detected); *—results of nasal swabs; Ct < 38 positive; Ct = 38–39–99 doubtful.

## Data Availability

The data presented in this study are available in this article and Supplementary Materials.

## Supplementary Materials

The following supporting information can be downloaded at: https://www.mdpi.com/article/10.3390/vaccines12060598/s1, Table S1: Clinical reaction score of all experimental animals after challenge with LSDV isolate “Mongolia/2021” (scoring according to the modified clinical score system of Carn and Kitching 1995.

## References

[1] Tuppurainen E.S.M., Venter E.H., Shisler J.L., Gari G., Mekonnen G.A., Juleff N., Lyons N.A., de Clercq K., Upton C., Bowden T.R. (2017). Review: Capripoxvirus Diseases: Current Status and Opportunities for Control. Transbound. Emerg. Dis..

[2] Biswas S., Noyce R.S., Babiuk L.A., Lung O., Bulach D.M., Bowden T.R., Boyle D.B., Babiuk S., Evans D.H. (2020). Extended Sequencing of Vaccine and Wild-Type Capripoxvirus Isolates Provides Insights into Genes Modulating Virulence and Host Range. Transbound. Emerg. Dis..

[3] Krotova A., Byadovskaya O., Shumilova I., van Schalkwyk A., Sprygin A. (2022). An In-Depth Bioinformatic Analysis of the Novel Recombinant Lumpy Skin Disease Virus Strains: From Unique Patterns to Established Lineage. BMC Genom..

[4] Van Schalkwyk A., Byadovskaya O., Shumilova I., Wallace D.B., Sprygin A. (2022). Estimating Evolutionary Changes between Highly Passaged and Original Parental Lumpy Skin Disease Virus Strains. Transbound. Emerg. Dis..

[5] Byadovskaya O., Prutnikov P., Shalina K., Babiuk S., Perevozchikova N., Korennoy F., Chvala I., Kononov A., Sprygin A. (2022). The Changing Epidemiology of Lumpy Skin Disease in Russia since the First Introduction from 2015 to 2020. Transbound. Emerg. Dis..

[6] Mazloum A., Van Schalkwyk A., Babiuk S., Venter E., Wallace D.B., Sprygin A. (2023). Lumpy Skin Disease: History, Current Understanding and Research Gaps in the Context of Recent Geographic Expansion. Front. Microbiol..

[7] Sudhakar S.B., Mishra N., Kalaiyarasu S., Jhade S.K., Hemadri D., Sood R., Bal G.C., Nayak M.K., Pradhan S.K., Singh V.P. (2020). Lumpy Skin Disease (LSD) Outbreaks in Cattle in Odisha State, India in August 2019: Epidemiological Features and Molecular Studies. Transbound. Emerg. Dis..

[8] Sprygin A., Pestova Y., Bjadovskaya O., Prutnikov P., Zinyakov N., Kononova S., Ruchnova O., Lozovoy D., Chvala I., Kononov A. (2020). Evidence of Recombination of Vaccine Strains of Lumpy Skin Disease Virus with Field Strains, Causing Disease. PLoS ONE.

[9] Wang Y., Zhao L., Yang J., Shi M., Nie F., Liu S., Wang Z., Huang D., Wu H., Li D. (2022). Analysis of Vaccine-like Lumpy Skin Disease Virus from Flies near the Western Border of China. Transbound. Emerg. Dis..

[10] Wang J., Xu Z., Wang Z., Li Q., Liang X., Ye S., Cheng K., Xu L., Mao J., Wang Z. (2022). Isolation, Identification and Phylogenetic Analysis of Lumpy Skin Disease Virus Strain of Outbreak in Guangdong, China. Transbound. Emerg. Dis..

[11] Calistri P., de Clercq K., Gubbins S., Klement E., Stegeman A., Cortiñas Abrahantes J., Marojevic D., Antoniou S., Broglia A. (2020). Lumpy Skin Disease Epidemiological Report IV: Data Collection and Analysis. EFSA J..

[12] Terrestrial Code Online Access—WOAH—World Organisation for Animal Health. https://www.woah.org/en/what-we-do/standards/codes-and-manuals/terrestrial-code-online-access/?id=169&L=1&htmfile=chapitre_lsd.htm.

[13] Haegeman A., De Leeuw I., Saduakassova M., van Campe W., Aerts L., Philips W., Sultanov A., Mostin L., de Clercq K. (2021). The Importance of Quality Control of Lsdv Live Attenuated Vaccines for Its Safe Application in the Field. Vaccines.

[14] Haegeman A., de Leeuw I., Mostin L., van Campe W., Aerts L., Venter E., Tuppurainen E., Saegerman C., de Clercq K. (2021). Comparative Evaluation of Lumpy Skin Disease Virus-Based Live Attenuated Vaccines. Vaccines.

[15] Gari G., Abie G., Gizaw D., Wubete A., Kidane M., Asgedom H., Bayissa B., Ayelet G., Oura C.A.L., Roger F. (2015). Evaluation of the Safety, Immunogenicity and Efficacy of Three Capripoxvirus Vaccine Strains against Lumpy Skin Disease Virus. Vaccine.

[16] Shumilova I., Krotova A., Nesterov A., Byadovskaya O., van Schalkwyk A., Sprygin A. (2022). Overwintering of Recombinant Lumpy Skin Disease Virus in Northern Latitudes, Russia. Transbound. Emerg. Dis..

[17] Nesterov A., Mazloum A., Byadovskaya O., Shumilova I., Van Schalkwyk A., Krotova A., Kirpichenko V., Donnik I., Chvala I., Sprygin A. (2022). Experimentally Controlled Study Indicates That the Naturally Occurring Recombinant Vaccine-like Lumpy Skin Disease Strain Udmurtiya/2019, Detected during Freezing Winter in Northern Latitudes, Is Transmitted via Indirect Contact. Front. Vet. Sci..

[18] Sprygin A., Sainnokhoi T., Gombo-Ochir D., Tserenchimed T., Tsolmon A., Byadovskaya O., Ankhanbaatar U., Mazloum A., Korennoy F., Chvala I. (2022). Genetic Characterization and Epidemiological Analysis of the First Lumpy Skin Disease Virus Outbreak in Mongolia, 2021. Transbound. Emerg. Dis..

[19] Sprygin A., Pestova Y., Prutnikov P., Kononov A. (2018). Detection of Vaccine-like Lumpy Skin Disease Virus in Cattle and *Musca domestica* L. Flies in an Outbreak of Lumpy Skin Disease in Russia in 2017. Transbound. Emerg. Dis..

[20] Aleksandr K., Olga B., David W.B., Pavel P., Yana P., Svetlana K., Alexander N., Vladimir R., Dmitriy L., Alexander S. (2020). Non-Vector-Borne Transmission of Lumpy Skin Disease Virus. Sci. Rep..

[21] Irons P.C., Tuppurainen E.S.M., Venter E.H. (2005). Excretion of Lumpy Skin Disease Virus in Bull Semen. Theriogenology.

[22] Carn V.M., Kitching R.P. (1995). The Clinical Response of Cattle Experimentally Infected with Lumpy Skin Disease (Neethling) Virus. Arch. Virol..

[23] Wolff J., Krstevski K., Beer M., Hoffmann B. (2020). Minimum Infective Dose of a Lumpy Skin Disease Virus Field Strain from North Macedonia. Viruses.

[24] Alexander S., Olga B., Svetlana K., Valeriy Z., Yana P., Pavel P., Aleksandr K. (2019). A Real-Time PCR Screening Assay for the Universal Detection of Lumpy Skin Disease Virus DNA. BMC Res. Notes.

[25] Wallace D.B., Mather A., Kara P.D., Naicker L., Mokoena N.B., Pretorius A., Nefefe T., Thema N., Babiuk S. (2020). Protection of Cattle Elicited Using a Bivalent Lumpy Skin Disease Virus-Vectored Recombinant Rift Valley Fever Vaccine. Front. Vet. Sci..

[26] Zhugunissov K., Bulatov Y., Orynbayev M., Kutumbetov L., Abduraimov Y., Shayakhmetov Y., Taranov D., Amanova Z., Mambetaliyev M., Absatova Z. (2020). Goatpox Virus (G20-LKV) Vaccine Strain Elicits a Protective Response in Cattle against Lumpy Skin Disease at Challenge with Lumpy Skin Disease Virulent Field Strain in a Comparative Study. Vet. Microbiol..

[27] World Health Organization (2022). World Health Statistics 2022: Monitoring Health for the SDGs, Sustainable Development Goals.

[28] Akther M., Akter S.H., Sarker S., Aleri J.W., Annandale H., Abraham S., Uddin J.M. (2023). Global Burden of Lumpy Skin Disease, Outbreaks, and Future Challenges. Viruses.

[29] Tuppurainen E.S.M., Alexandrov T., Beltrán-Alcrudo D. (2017). Lumpy Skin Disease Field Manual—A Manual for Veterinarians.

[30] Namazi F., Khodakaram Tafti A. (2021). Lumpy Skin Disease, an Emerging Transboundary Viral Disease: A Review. Vet. Med. Sci..

[31] Matsiela M.S., Naicker L., Dibakwane V.S., Ntombela N., Khoza T., Mokoena N. (2022). Improved Safety Profile of Inactivated Neethling Strain of the Lumpy Skin Disease Vaccine. Vaccine X.

[32] Tuppurainen E.S.M., Antoniou S.E., Tsiamadis E., Topkaridou M., Labus T., Debeljak Z., Plavšić B., Miteva A., Alexandrov T., Pite L. (2020). Field Observations and Experiences Gained from the Implementation of Control Measures against Lumpy Skin Disease in South-East Europe between 2015 and 2017. Prev. Vet. Med..

[33] Kitching R.P. (2003). Vaccines for Lumpy Skin Disease, Sheep Pox and Goat Pox. Dev. Biol..

[34] Tuppurainen E.S.M., Pearson C.R., Bachanek-Bankowska K., Knowles N.J., Amareen S., Frost L., Henstock M.R., Lamien C.E., Diallo A., Mertens P.P.C. (2014). Characterization of Sheep Pox Virus Vaccine for Cattle against Lumpy Skin Disease Virus. Antivir. Res..

[35] Ben-Gera J., Klement E., Khinich E., Stram Y., Shpigel N.Y. (2015). Comparison of the Efficacy of Neethling Lumpy Skin Disease Virus and X10RM65 Sheep-Pox Live Attenuated Vaccines for the Prevention of Lumpy Skin Disease—The Results of a Randomized Controlled Field Study. Vaccine.

[36] Mathan Kum S. (2011). An Outbreak of Lumpy Skin Disease in a Holstein Dairy Herd in Oman: A Clinical Report. Asian J. Anim. Vet. Adv..

[37] Hakobyan V., Sargsyan K., Kharatyan S., Elbakyan H., Sargsyan V., Markosyan T., Vardanyan T., Badalyan M., Achenbach J.E. (2023). The Serological Response in Cattle Following Administration of a Heterologous Sheep Pox Virus Strain Vaccine for Protection from Lumpy Skin Disease; Current Situation in Armenia. Vet. Sci..

[38] Uzar S., Sarac F., Gulyaz V., Enul H., Yılmaz H., Turan N. (2022). Comparison and Efficacy of Two Different Sheep Pox Vaccines Prepared from the Bakırköy Strain against Lumpy Skin Disease in Cattle. Clin. Exp. Vaccine Res..

[39] Klement E., Broglia A., Antoniou S.E., Tsiamadis V., Plevraki E., Petrović T., Polaček V., Debeljak Z., Miteva A., Alexandrov T. (2020). Neethling Vaccine Proved Highly Effective in Controlling Lumpy Skin Disease Epidemics in the Balkans. Prev. Vet. Med..

[40] Hamdi J., Boumart Z., Daouam S., El Arkam A., Bamouh Z., Jazouli M., Tadlaoui K.O., Fihri O.F., Gavrilov B., El Harrak M. (2020). Development and Evaluation of an Inactivated Lumpy Skin Disease Vaccine for Cattle. Vet. Microbiol..

[41] Abutarbush S.M., Hananeh W.M., Ramadan W., Al Sheyab O.M., Alnajjar A.R., Al Zoubi I.G., Knowles N.J., Bachanek-Bankowska K., Tuppurainen E.S.M. (2016). Adverse Reactions to Field Vaccination Against Lumpy Skin Disease in Jordan. Transbound. Emerg. Dis..

[42] Abdelwahab M.G., Khafagy H.A., Moustafa A.M., Saad M.A. (2016). Evaluation of Humoral and Cell-Mediated Immunity of Lumpy Skin Disease Vaccine Prepared from Local Strainin Calves and Its Related to Maternal Immunity Evaluation of Humoral and Cell-Mediated Immunity of Lumpy Skin Disease Vaccine Prepared from Local in Calves and Its Related to Maternal Immunity. J. Am. Sci..

[43] Weiss K.E. (1968). Lumpy Skin Disease Virus. Cytomegaloviruses. Rinderpest Virus. Lumpy Skin Disease Virus.

[44] Katsoulos P.D., Chaintoutis S.C., Dovas C.I., Polizopoulou Z.S., Brellou G.D., Agianniotaki E.I., Tasioudi K.E., Chondrokouki E., Papadopoulos O., Karatzias H. (2018). Investigation on the Incidence of Adverse Reactions, Viraemia and Haematological Changes Following Field Immunization of Cattle Using a Live Attenuated Vaccine against Lumpy Skin Disease. Transbound. Emerg. Dis..

[45] Bedeković T., Šimić I., Krešić N., Lojkić I. (2018). Detection of Lumpy Skin Disease Virus in Skin Lesions, Blood, Nasal Swabs and Milk Following Preventive Vaccination. Transbound. Emerg. Dis..

[46] Lojkić I., Šimić I., Krešić N., Bedeković T. (2018). Complete Genome Sequence of a Lumpy Skin Disease Virus Strain Isolated from the Skin of a Vaccinated Animal. Genome Announc..

[47] Tuppurainen E.S.M., Venter E.H., Coetzer J.A.W. (2005). The Detection of Lumpy Skin Disease Virus in Samples of Experimentally Infected Cattle Using Different Diagnostic Techniques. Onderstepoort J. Vet. Res..

[48] Möller J., Moritz T., Schlottau K., Krstevski K., Hoffmann D., Beer M., Hoffmann B. (2019). Experimental Lumpy Skin Disease Virus Infection of Cattle: Comparison of a Field Strain and a Vaccine Strain. Arch. Virol..

[49] Office International Des Epizooties (OIE) (2016). Chapter 2.4.13.—Lumpy Skin Disease. Terrestrial Manual.

[50] Babiuk S., Bowden T.R., Parkyn G., Dalman B., Manning L., Neufeld J., Embury-Hyatt C., Copps J., Boyle D.B. (2008). Quantification of Lumpy Skin Disease Virus Following Experimental Infection in Cattle. Transbound. Emerg. Dis..

[51] Kumar N., Barua S., Kumar R., Khandelwal N., Kumar A., Verma A., Singh L., Godara B., Chander Y., Kumar G. (2023). Evaluation of the Safety, Immunogenicity and Efficacy of a New Live-Attenuated Lumpy Skin Disease Vaccine in India. Virulence.

[52] Gaber A., Rouby S., Elsaied A., El-Sherif A. (2022). Assessment of Heterologous Lumpy Skin Disease Vaccine-Induced Immunity in Pregnant Cattle Vaccinated at Different Times of Gestation Period and Their Influence on Maternally Derived Antibodies. Vet. Immunol. Immunopathol..

[53] Abdallah F.M., El Damaty H.M., Kotb G.F. (2018). Sporadic Cases of Lumpy Skin Disease among Cattle in Sharkia Province, Egypt: Genetic Characterization of Lumpy Skin Disease Virus Isolates and Pathological Findings. Vet. World.

[54] Bamouh Z., Hamdi J., Fellahi S., Khayi S., Jazouli M., Tadlaoui K.O., Fihri O.F., Tuppurainen E., Elharrak M. (2021). Investigation of Post Vaccination Reactions of Two Live Attenuated Vaccines against Lumpy Skin Disease of Cattle. Vaccines.

[55] Wolff J., Moritz T., Schlottau K., Hoffmann D., Beer M., Hoffmann B. (2020). Development of a Safe and Highly Efficient Inactivated Vaccine Candidate against Lumpy Skin Disease Virus. Vaccines.

[56] Mosad S.M., Rasheed N., Ali H.S., El-Khabaz K.A.S., Shosha E.A.M., El-Diasty M. (2021). Incidence of Lumpy Skin Disease Virus with Its Characterization in Vaccinated Pregnant Holstein Cows in Dakahlia Governorate, Egypt. Ger. J. Vet. Res..

[57] Mikhael C., Ibrahim M., Awad M., Soliman S., Michael A. (2014). Comparative Study on the Efficiency of Some Capripox Vaccines in Protection of Cattle against Lumpy Skin Disease. Suez Canal Vet. Med. J. SCVMJ.

[58] Tscharke D.C., Woo W.-P., Sakala I.G., Sidney J., Sette A., Moss D.J., Bennink J.R., Karupiah G., Yewdell J.W. (2006). Poxvirus CD8 + T-Cell Determinants and Cross-Reactivity in BALB/c Mice. J. Virol..

[59] McLoughlin K.E., Correia C.N., Browne J.A., Magee D.A., Nalpas N.C., Rue-Albrecht K., Whelan A.O., Villarreal-Ramos B., Vordermeier H.M., Gormley E. (2021). RNA-Seq Transcriptome Analysis of Peripheral Blood from Cattle Infected With Mycobacterium Bovis Across an Experimental Time Course. Front. Vet. Sci..

