# Supplementary material for: An Attenuated Vaccine Virus of the Neethling Lineage Protects Cattle against the Virulent Recombinant Vaccine-like Isolate of the Lumpy Skin Disease Virus Belonging to the Currently Established Cluster 2.5
Source: Vaccines (Basel). 2024 May 30;12(6):598. doi: 10.3390/vaccines12060598 (PMC11209201; doi:10.3390/vaccines12060598)
Supplement: Supplementary file 1 [file vaccines-12-00598-s001.zip › vaccines-2969224-supplementary.pdf]

Table S1. Clinical reaction score of all experimental animals after challenge with LSDV isolate "Mongolia/2021" (scoring according to the modified clinical score system of Carn and Kitching 1995.

Animal	Clinical signs	Clinical reaction score	Type of infection
**Challenge day**			
1-IV	No clinical signs. Normal temperature.	0	intravenously
2-IV	No clinical signs. Normal temperature.	0	
3-IV	No clinical signs. Normal temperature.	0	
4-IV	No clinical signs. Normal temperature.	0	
5-IV	No clinical signs. Normal temperature.	0	
6-IV-C	No clinical signs. Normal temperature.	0	
7-IV-C	No clinical signs. Normal temperature.	0	
8-IV	No clinical signs. Normal temperature.	0	
1-SC	No clinical signs. Normal temperature.	0	titration
2-SC	No clinical signs. Normal temperature.	0	
3-SC	No clinical signs. Normal temperature.	0	
4-SC	No clinical signs. Normal temperature.	0	
5-SC	No clinical signs. Normal temperature.	0	
6-SC-C	No clinical signs. Normal temperature.	0	
7-SC-C	No clinical signs. Normal temperature.	0	
8-SC	No clinical signs. Normal temperature.	0	
**Day 1 after challenge**			
1-IV	No clinical signs. Normal temperature.	0	intravenously
2-IV	No clinical signs. Normal temperature.	0	
3-IV	No clinical signs. Normal temperature.	0	
4-IV	No clinical signs. Normal temperature.	0	
5-IV	No clinical signs. Normal temperature.	0	
6-IV-C	No clinical signs. Normal temperature.	0	
7-IV-C	No clinical signs. Normal temperature.	0	
8-IV	No clinical signs. Normal temperature.	0	
1-SC	No clinical signs. Normal temperature.	0	titration
2-SC	No clinical signs. Normal temperature.	0	
3-SC	No clinical signs. Normal temperature.	0	
4-SC	No clinical signs. Normal temperature.	0	
5-SC	No clinical signs. Normal temperature.	0	
6-SC-C	No clinical signs. Normal temperature.	0	
7-SC-C	No clinical signs. Normal temperature.	0	
8-SC	No clinical signs. Normal temperature.	0	
**Day 3 after challenge**			
1-IV	No clinical signs. Normal temperature.	0	intravenously
2-IV	No clinical signs. Normal temperature.	0	
3-IV	No clinical signs. Normal temperature.	0	
4-IV	No clinical signs. Normal temperature.	0	
5-IV	No clinical signs. Normal temperature.	0	
6-IV-C	No clinical signs. Normal temperature.	0	
7-IV-C	No clinical signs. Normal temperature.	0	
8-IV	No clinical signs. Normal temperature.	0	
1-SC	No clinical signs. Normal temperature.	0	titration
2-SC	No clinical signs. Normal temperature.	0	
3-SC	No clinical signs. Normal temperature.	0	

4-SC	No clinical signs. Normal temperature.	0	
5-SC	No clinical signs. Normal temperature.	0	
6-SC-C	No clinical signs. Normal temperature.	0	
7-SC-C	No clinical signs. Normal temperature.	0	
8-SC	No clinical signs. Normal temperature.	0	
**Day 4 after challenge**			
1-IV	No clinical signs. Normal temperature.	0	
2-IV	No clinical signs. Normal temperature.	0	
3-IV	No clinical signs. Normal temperature.	0	
4-IV	No clinical signs. Normal temperature.	0	
5-IV	No clinical signs. Normal temperature.	0	intravenously
6-IV-C	Mild local reaction at the site of injection of the initial dilutions of virus (< 6 cm diameter), lymphadenopathy, temperature 39.9^{0}C.	3	
7-IV-C	Mild local reaction at the site of injection of the initial dilutions of virus (< 6 cm diameter), lymphadenopathy, temperature 39.8^{0}C.	3	
8-IV	No clinical signs. Normal temperature.	0	
1-SC	No clinical signs. Normal temperature.	0	
2-SC	No clinical signs. Normal temperature.	0	
3-SC	No clinical signs. Normal temperature.	0	
4-SC	No clinical signs. Normal temperature.	0	
5-SC	No clinical signs. Normal temperature.	0	titration
6-SC-C	Mild local reaction at the site of injection of the initial dilutions of virus (< 5 cm diameter), without lymphadenopathy, temperature 39.8^{0}C.	2	
7-SC-C	No clinical signs. Normal temperature.	0	
8-SC	No clinical signs. Normal temperature.	0	
**Day 6 after challenge**			
1-IV	No clinical signs. Normal temperature.	0	
2-IV	No clinical signs. Normal temperature.	0	
3-IV	No clinical signs. Normal temperature.	0	
4-IV	No clinical signs. Normal temperature.	0	
5-IV	No clinical signs. Normal temperature.	0	intravenously
6-IV-C	Severe local reaction: heat, pain, oedema, lesion > 6 cm diameter. Enlarged prescapular lymph, temperature 40.2^{0}C.	5	
7-IV-C	Moderate local reaction: > 6 cm in diameter, with some heat, pain and oedema, lymphadenopathy, temperature 40^{0}C.	4	
8-IV	No clinical signs. Normal temperature.	0	
1-SC	No clinical signs. Normal temperature.	0	
2-SC	No clinical signs. Normal temperature.	0	
3-SC	No clinical signs. Normal temperature.	0	
4-SC	No clinical signs. Normal temperature.	0	
5-SC	No clinical signs. Normal temperature.	0	titration
6-SC-C	Mild local reaction at the site of injection of the initial dilutions of virus (< 5 cm diameter), without lymphadenopathy, temperature 40^{0}C.	2	
7-SC-C	Mild local reaction, temperature 39,7^{0}C.	1	
8-SC	No clinical signs. Normal temperature.	0	
**Day 9 after challenge**			
1-IV	No clinical signs. Normal temperature.	0	
2-IV	No clinical signs. Normal temperature.	0	
3-IV	No clinical signs. Normal temperature.	0	intravenously
4-IV	No clinical signs. Normal temperature.	0	
5-IV	No clinical signs. Normal temperature.	0	

6-IV-C	Generalisation with few secondary nodules, severe lymphadenopathy, no systemic disturbance, temperature 41^{0}C.	7	
7-IV-C	Severe local reaction at inoculation site: heat, pain, oedema, lesion > 6 cm in diameter. Severe lymphadenopathy, temperature 40.4^{0}C.	6	
8-IV	No clinical signs. Normal temperature.	0	
1-SC	No clinical signs. Normal temperature.	0	titration
2-SC	No clinical signs. Normal temperature.	0	
3-SC	No clinical signs. Normal temperature.	0	
4-SC	No clinical signs. Normal temperature.	0	
5-SC	No clinical signs. Normal temperature.	0	
6-SC-C	Moderate local reaction: > 6 cm in diameter, with some heat, pain and oedema, lymphadenopathy, temperature 40.1^{0}C.	4	
7-SC-C	Severe local reaction: heat, pain, oedema, lesion > 6 cm diameter. Enlarged prescapular lymph, temperature 40^{0}C.	5	
8-SC	No clinical signs. Normal temperature.	0	
**Day 12 after challenge**			
1-IV	No clinical signs. Normal temperature.	0	intravenously
2-IV	No clinical signs. Normal temperature.	0	
3-IV	No clinical signs. Normal temperature.	0	
4-IV	No clinical signs. Normal temperature.	0	
5-IV	No clinical signs. Normal temperature.	0	
6-IV-C	Generalisation with few secondary nodules, severe lymphadenopathy, no systemic disturbance, temperature 41^{0}C.	7	
7-IV-C	Severe local reaction at inoculation site: heat, pain, oedema, lesion > 6 cm in diameter. Severe lymphadenopathy, temperature 41^{0}C.	6	
8-IV	No clinical signs. Normal temperature.	0	
1-SC	No clinical signs. Normal temperature.	0	titration
2-SC	No clinical signs. Normal temperature.	0	
3-SC	No clinical signs. Normal temperature.	0	
4-SC	No clinical signs. Normal temperature.	0	
5-SC	No clinical signs. Normal temperature.	0	
6-SC-C	Severe local reaction: heat, pain, oedema, lesion > 6 cm diameter. Enlarged prescapular lymph, temperature 40.2^{0}C.	5	
7-SC-C	Severe local reaction at inoculation site: heat, pain, oedema, lesion > 6 cm in diameter. Severe lymphadenopathy, temperature 40.5^{0}C.	6	
8-SC	No clinical signs. Normal temperature.	0	
**Day 15 after challenge**			
1-IV	No clinical signs. Normal temperature.	0	intravenously
2-IV	No clinical signs. Normal temperature.	0	
3-IV	No clinical signs. Normal temperature.	0	
4-IV	No clinical signs. Normal temperature.	0	
5-IV	No clinical signs. Normal temperature.	0	
6-IV-C	Generalisation with many secondary lesions and severe lymphadenpathy. No systemic disturbance, temperature 41.2^{0}C.	8	
7-IV-C	Generalisation with few secondary nodules, severe lymphadenopathy, no systemic disturbance, temperature 40.5^{0}C.	7	
8-IV	No clinical signs. Normal temperature.	0	titration
1-SC	No clinical signs. Normal temperature.	0	
2-SC	No clinical signs. Normal temperature.	0	
3-SC	No clinical signs. Normal temperature.	0	
4-SC	No clinical signs. Normal temperature.	0	
5-SC	No clinical signs. Normal temperature.	0	

6-SC-C	Severe local reaction at inoculation site: heat, pain, oedema, lesion > 6 cm in diameter. Severe lymphadenopathy, temperature 40.5^{0}C.	6	
7-SC-C	Severe local reaction at inoculation site: heat, pain, oedema, lesion > 6 cm in diameter. Severe lymphadenopathy, temperature 40.4^{0}C.	6	
8-SC	No clinical signs. Normal temperature.	0	
**Day 18 after challenge**			
1-IV	No clinical signs. Normal temperature.	0	intravenously
2-IV	No clinical signs. Normal temperature.	0	
3-IV	No clinical signs. Normal temperature.	0	
4-IV	No clinical signs. Normal temperature.	0	
5-IV	No clinical signs. Normal temperature.	0	
6-IV-C	Severe generalisation with depression. Numerous secondary nodules, severe lymphadenopathy, conjunctivitis and rhinitis, temperature 41.5^{0}C.	9	
7-IV-C	Generalisation with few secondary nodules, severe lymphadenopathy, no systemic disturbance, temperature 40.3^{0}C.	7	
8-IV	No clinical signs. Normal temperature.	0	
1-SC	No clinical signs. Normal temperature.	0	titration
2-SC	No clinical signs. Normal temperature.	0	
3-SC	No clinical signs. Normal temperature.	0	
4-SC	No clinical signs. Normal temperature.	0	
5-SC	No clinical signs. Normal temperature.	0	
6-SC-C	Generalisation with many secondary lesions and severe lymphadenpathy. No systemic disturbance, temperature 41^{0}C.	8	
7-SC-C	Severe local reaction at inoculation site: heat, pain, oedema, lesion > 6 cm in diameter. Severe lymphadenopathy, temperature 40.5^{0}C.	6	
8-SC	No clinical signs. Normal temperature.	0	
**Day 20 after challenge**			
1-IV	No clinical signs. Normal temperature.	0	intravenously
2-IV	No clinical signs. Normal temperature.	0	
3-IV	No clinical signs. Normal temperature.	0	
4-IV	No clinical signs. Normal temperature.	0	
5-IV	No clinical signs. Normal temperature.	0	
6-IV-C	Severe generalisation with depression. Numerous secondary nodules, severe lymphadenopathy, conjunctivitis and rhinitis, temperature 41.3^{0}C.	9	
7-IV-C	Generalisation with many secondary lesions and severe lymphadenpathy. No systemic disturbance, temperature 40.5^{0}C.	8	
8-IV	No clinical signs. Normal temperature.	0	
1-SC	No clinical signs. Normal temperature.	0	titration
2-SC	No clinical signs. Normal temperature.	0	
3-SC	No clinical signs. Normal temperature.	0	
4-SC	No clinical signs. Normal temperature.	0	
5-SC	No clinical signs. Normal temperature.	0	
6-SC-C	Generalisation with many secondary lesions and severe lymphadenpathy. No systemic disturbance, temperature 40.7^{0}C.	8	
7-SC-C	Generalisation with few secondary nodules, severe lymphadenopathy, no systemic disturbance, temperature 40.7^{0}C.	7	
8-SC	No clinical signs. Normal temperature.	0	
**Day 22 after challenge**			
1-IV	No clinical signs. Normal temperature.	0	intravenously
2-IV	No clinical signs. Normal temperature.	0	

3-IV	No clinical signs. Normal temperature.	0	
4-IV	No clinical signs. Normal temperature.	0	
5-IV	No clinical signs. Normal temperature.	0	
6-IV-C	Severe generalisation. Numerous secondary nodules, 0.5-5 cm diameter, with oedema, hyperaemia and pain. Severe lymphadenopathy, conjunctivitis, rhinitis, severe debility and inappetence, temperature 41.5^{0}C.	10	
7-IV-C	Severe generalisation. Numerous secondary nodules, 0.5-5 cm diameter, with oedema, hyperaemia and pain. Severe lymphadenopathy, conjunctivitis, rhinitis, severe debility and inappetence, temperature 41.3^{0}C.	10	
8-IV	No clinical signs. Normal temperature.	0	
1-SC	No clinical signs. Normal temperature.	0	titration
2-SC	No clinical signs. Normal temperature.	0	
3-SC	No clinical signs. Normal temperature.	0	
4-SC	No clinical signs. Normal temperature.	0	
5-SC	No clinical signs. Normal temperature.	0	
6-SC-C	Severe generalisation with depression. Numerous secondary nodules, severe lymphadenopathy, conjunctivitis and rhinitis, temperature 41.1^{0}C.	9	
7-SC-C	Generalisation with few secondary nodules, severe lymphadenopathy, no systemic disturbance, temperature 40.5^{0}C.	7	
8-SC	No clinical signs. Normal temperature.	0	
**All animals were euthanized.**			
